# Annotations

**Published:** 1894-11-24

**Authors:** 


### Annotations.

### The Dangers of Strong Tea.

That the continuous use of strong and improperly infused tea is seriously harmful has long been known to experienced medical practitioners. A case reported from New York well illustrates some of the dangers of the practice. The person of whom the facts are recorded was a healthy young man of thirty. He was a total abstainer and a non-smoker. In November, 1892, having been up to that time a non-tea-drinker, he commenced the habit of taking half a pint of strong tea at noon, and a second half-pint at four o'clock. Those quantities he gradually increased until he was taking three full pints a day—a ridiculous quantity it must be admitted. After continuing for six weeks he began to experience a "mild tingling" in the right hand, which speedily extended up the forearm. Small "blebs" appeared on the fingers, the skin of the body became unusually sensitive, and the heart's action feeble and irregular. The tea-drinking was thereupon stopped, and in six weeks, with suitable treatment, the patient became quite well. About fifteen months later, having probably almost forgotten his former experience, the young man returned to his passion for strong tea. In less than a month his former symptoms, all more severe, and some new ones, began to appear. A dull, boring, burning pain was felt in the right wrist, and this soon extended up the arm and across the shoulder blade to the spine. The brachial plexus became involved, and pressure in every part of the axillary space produced intense pain, whilst over the median nerve pressure could not be tolerated at all. If the hand were placed in warm water of not more than 50 deg. F. the pain produced was so great that it had to be withdrawn. These and many other symptoms were so manifestly due to the strong tea that it was again given up, and again a cure resulted. The moral of the story is that, however delightful a stimulant good tea, properly infused and taken in moderation, may be, its excessive use should be avoided, especially by those who have once shown susceptibility to its influence.

### The Treatment of Intestinal Obstruction.

At a recent meeting of the Medical Society an interesting point was discussed in reference to the question whether to do enterectomy and make an intestinal anastomosis as part of the operation for relieving obstruction of the bowe's, or to content oneself at the time with making an artificial anus as an operation of emergency, and doing the rest afterwards. Mr. Harrison Cripps and Mr. Herbert Allingham both agreed that, for many reasons, it was best to delay the more delicate operation until it could be done under the best conditions, merely making in the emergency such an artificial anus as would relieve the urgent condition of obstruction. Especial stress was laid on the fact that by delay one could ensure that the bowel should be fairly empty, so that the necessity of uniting a distended and paralysed gut with an empty and contracted portion should be done away with, and that by doing the operation when the bowel was in a state of rest, and the patient free from the toxic poisoning arising from the obstruction, there was much greater probability of proper union of the opposed surfaces, whatever method of anastomosis might be adopted. All this is interesting to the hospital surgeon, but it is infinitely more so to the general practitioner. So long as surgical interference in cases of obstruction meant elaborate methods, with bone-plates, buttons, and other mechanical appliances, the general practitioner might well hesitate to interfere. But to make an artificial anus, and then hand the case over to the specialist is a comparatively simple matter, and it must be remembered that an operation, done with the definite aim of opening the first presenting distended bowel, is a far less serious affair than opening the bowel as a *dernier resort* after divers attempts to relieve an obstruction. The moral then for the general practitioner, often situated far from efficient help, is to make an artificial anus at the presenting bowel, whatever it may be, and not to interfere further, leaving the rest for the specialist in such matters.

### The Trade in Remedies for Diphtheria.

As will be seen from an article in another column, we feel far from satisfied that we have attained finality in serum therapeutics, nevertheless, the evidence of the utility of such antitoxins as have already been produced is so striking that it is of great importance that those who make use of them should have some sort of guarantee of the quality of the stuff they are going to inject. Writing on this subject so far back as October 20th, the *British Medical Journal* said: "It would be a great scandal were the market to be flooded with competing brands of such a substance, produced by different firms at 'cutting' prices, and if the public when in peril from diphtheria were to be exposed to the added danger of being injected with bad antitoxins." It seems as if the scandal prophesied were already rising into being, for we find a trade journal devoting the greater part of this month's issue to a laudation of Aronson's antitoxin as opposed to that produced by Roux in France, or Ruffer in England, and to ridicule of the British Institute of Preventive Medicine for presuming to undertake the supply of such a material. In our view the argument cuts the other way. It is at present open to any bacteriologist who can afford to buy a few horses to flood the market with a substance which may or may not be antitoxin. If the chemist who is the agent for one antitoxin devotes his journal to its defence, other chemists who run journals will soon enter the field and push their own productions, and a pretty war we shall have. The statement that the British Institute would be able to produce antitoxin at less than the present price has already elicited the counter statement that Aronson's material will also come down in cost, together with the sneer that the British Institute estimates are "the hypothetical calculations of inexperienced philanthropists." All this is very degrading, and only enforces the contention that the production of such a remedy should not fall into the hands of chemists, but should be undertaken by some institution which would give to its product a stamp of authority.

